# Dengue Specific Immunoglobulin A Antibody is Present in Urine and Associated with Disease Severity

**DOI:** 10.1038/srep27298

**Published:** 2016-06-02

**Authors:** Hui Zhao, Shuang Qiu, Wen-Xin Hong, Ke-Yu Song, Jian Wang, Hui-Qin Yang, Yong-Qiang Deng, Shun-Ya Zhu, Fu-Chun Zhang, Cheng-Feng Qin

**Affiliations:** 1 Department of Virology, State Key Laboratory of Pathogen and Biosecurity, Beijing Institute of Microbiology and Epidemiology, Beijing 100071, People’s Republic of China; 2 Guangzhou Eighth People’s Hospital, Guangzhou Medical University, Guangzhou 510060, People’s Republic of China

## Abstract

The kinetics of dengue virus (DENV)-specific IgA antibody in urine and the potential correlation with disease severity remain elusive. In this study, 262 serial urine samples from 78 laboratory-confirmed patients were assayed by a commercial immunoglobulin A (IgA) kit against DENV. All cases were classified into dengue fever (DF) and severe dengue (SD) according to the 2009 WHO/TDR guideline. The total positive rate of IgA in urine was 59%. DENV-specific IgA was detected in urine from day 2 to day 13 after the onset of illness in DF patients; While for SD patients, anti-DENV IgA could be detected till day 14. The positive rate of IgA in patients with secondary infection was higher than that in patients with primary infection. Importantly, during 4–7 days after the onset of illness, the IgA positive rate of SD patients was significantly higher than that of DF patients. Especially, the intensity of IgA signal in SD patients was obviously stronger than that in DF patient at the recovery stage. Overall, our results suggested that the existence of DENV-specific IgA antibodies in urine might be a warning sign for the severity of disease and its measurement might provide valuable guidance for proper patient management.

Dengue has constituted a public health emergency of international concern due to the rapid spread and domination in the world. Dengue has a wide spectrum of clinical presentations, often with unpredictable clinical evolution and outcome^1^. The four serotypes (1~4) of dengue virus (DENV) can cause either mild or severe clinical manifestations, and the latter is generally associated with sequential secondary heterotypic DENV infection. According to the 2009 WHO/TDR dengue guidelines for diagnosis, treatment, prevention and control, dengue can be further classified with dengue fever (DF) and severe dengue (SD)^2^. Recently, several countries have licensed Dengvaxia from Sanofi Pateur for the prevention of dengue. Early laboratory diagnosis should be of high value because some patients progress from mild to severe disease and sometimes to death over a short period^3^.

Serological assays are now widely used for dengue diagnosis. Dengue-specific IgM and IgG capture enzyme linked immunosorbent assays (ELISAs) are the most common serological assays and dozens of commercial kits have been approved for clinical use in various countries^4^. Recently, DENV-specific IgA antibodies were detected in serum and saliva of dengue patients^5^,^6^. Moreover, several studies have reported a higher IgA response in serum in secondary infection cases^7^,^8^,^9^, and the levels of IgA in serum might be correlated with the severity of disease^10^,^11^. In addition, a commercial dengue IgA rapid test has been developed based on immunochromatographic technology, which was more sensitive and specific as compared to reference ELISA^12^,^13^,^14^,^15^.

Recently, urine is becoming increasingly popular as a noninvasive approach for the laboratory diagnosis of dengue cases when blood samples are difficult to obtain^16^,^17^,^18^,^19^. However, it has not been evaluated whether the detection of urinary IgA is an alternative method used for dengue diagnosis. Vasquez *et al.* has reported the kinetics of IgA in serum, saliva and urine samples from adult patients with dengue infection^20^. However, the kinetics and role of DENV-specific IgA in urine samples from patients with different clinical manifestations and infection type remain largely unknown. The aim of this study is to characterize the DENV-IgA antibody profiles in urine samples from DF and SD patients, and to clarify the potential relationship between IgA and the severity of disease.

## Results

### Characteristics of the enrolled patients

Table 1 summarizes the characteristics of all enrolled patients. Of all patients, 53 were classified as DF and 25 as SD according to the criteria defined by 2009 WHO/TDR guidelines^2^. The proportion of dengue for males was similar to females (*P* = 0.321). The median age of patients with DF was 38.8 ± 16.7 years old, while 61.4 ± 20.7 for SD, which implied that the most affected cases were old people in SD cases (*P* < 0.001). Among the SD patients, 56.0% were diagnosed as secondary infections. In contrast, only 15.1% of the DF patients were identified as secondary infections. The ratio of secondary infection in patients with SD was significantly higher than patients with DF (*P* < 0.001). In addition, the ratio of acute kidney injury (AKI) in patients with SD was significantly higher than patients with DF (*P* < 0.001). DENV-specific real-time RT-PCR assay revealed that all four DENV serotypes were found in the acute sera from enrolled patients, and 62 patients were infected with DENV serotype 1. Two DF cases and three SD cases, that were negative for DENV nucleic acid detection, were confirmed with NS1 antigen examination for DENV.

### Detection of DENV-IgA in urine samples

**Figure 1:**
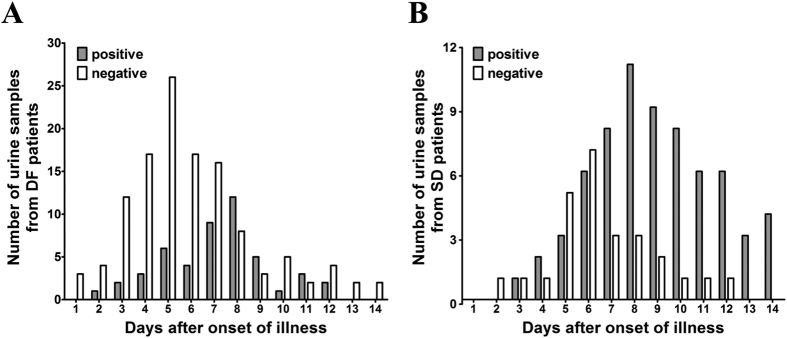
Distribution of IgA-positive and IgA-negative urine samples from DF (**A**) and SD (**B**) patients over the 14-days period.

None of the 20 urine samples collected from healthy donors was reactive for DENV-specific IgA. Of the 78 patients, only 46 (59.0%) were IgA positive, including 24 DF patients and 22 SD patients (Table 2). The kinetics of DENV-specific IgA antibodies in urine was depicted in Fig. 1. Of DF patients (Fig. 1A), anti-DENV IgA was detected from day 2 till day 13 after the onset of the illness. While for SD patients (Fig. 1B), anti-DENV IgA was positive from day 3 to day 14. The detailed information of each patient was listed in Table S1. Of the 22 patients with secondary infection, 59.1% was positive for DENV-specific IgA in urine. In contrast, for patients with primary infection, only 23.2% (13/56) was positive. The positive rate of IgA in patients with secondary infection was significantly higher than patients with primary infection (*P* = 0.0038) (Table 3).

### Comparison of the positive rate of DENV-IgA detection between DF and SD groups

Table 2 shows the positive rate of DENV-IgA detection among DF and SD patients recruited in the study. There were 53 DF and 25 SD cases totally. Only 24 (45.3%) DF cases were positive for IgA, whereas 22 (88%) SD cases were positive. During the different phase of the illness, the positive rate of IgA in the urine of SD patients was higher than that in DF patients. The proportion of patients distributed across different time limits was not well balanced (Table S2), the data during 4–7 days after the onset of the illness were used to analyze to evaluate the association between IgA positive rate and disease severity. Statistical analysis using Fisher exact tests demonstrated that there was a significant increase of IgA positive rate in the urine from SD patients compared with DF patients (*P* = 0.017). These data implied that urinary IgA might be a significant marker for the diagnosis of SD.

**Figure 2:**
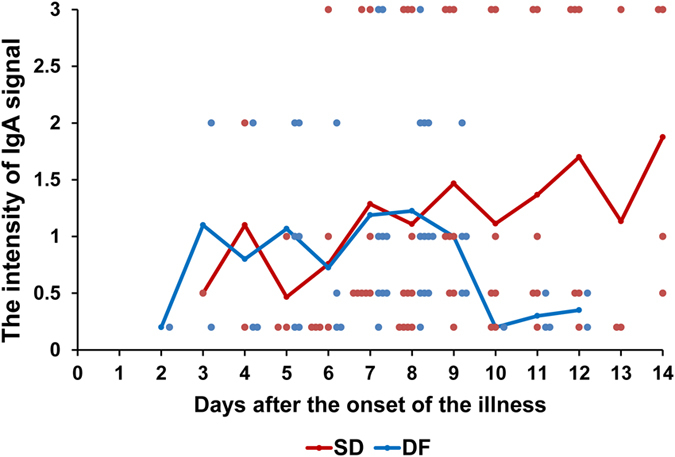
Kinetics of the intensity of IgA signal in urine samples from DF and SD patients over the 14-days period. The intensity of IgA signal in the urine over time was shown for 24 patients with DF and 22 with SD. 48 data points were from the DF group and 68 data points from the SD group.

Furthermore, we analyzed the difference in terms of the intensity of IgA signal between DF and SD cases. As shown in Fig. 2, there was no obvious difference between SD and DF patients at the early stage of disease (Day 2 to day 8). However, the IgA signal intensity in SD patients was obviously higher than that in DF group since day 8 post disease onset. Moreover, detail analysis for serial samples from the same patients revealed high consistence and strong signal intensity (>1). These observations confirmed that the higher level of IgA signal is relevant to progress to serious dengue disease.

## Discussion

We reported here the first evidence of correlation between urinary IgA and the severity of dengue disease. Our results showed the IgA positive rate of SD patients was significantly higher than that of DF patients during 4–7 days after the onset of illness. Furthermore, the intensity of IgA signal in SD patients was obviously stronger than that in DF patients at the recovery stage. This finding showed that the presence of IgA in urine may be used as a warning sign for the development of SD, especially when detected during 4–7 days of disease onset. Urine is a body fluid with low concentration of proteins derived from plasma. However, abundant proteins occur in the urine due to the enhancement of glomerular filtration membrane permeability when kidney is damaged. It has been known that AKI is a potential complication of SD^21^. The prevalence of AKI was 4.9% among 81 adults with dengue hemorrhagic fever (DHF)^22^, and 60% in fatal DHF in Taiwan^15^. In India, AKI was observed in 10.8% of 223 patients with DF, and was associated with an increased mortality^23^. A recent report showed that DHF-related AKI caused a high mortality rate of 64.0% in Thailand^24^. In the present study, 64% of SD patients had AKI, which was a possible factor resulting in a significant increase of IgA antibody in the urine, compared to that in DF patients (21% with AKI). Very recently, Upadhaya reported a dengue case with AKI, and renal biopsy demonstrated IgA deposits in the mesangium^25^.

Generally, secondary heterotypic infection is known as a risk factor for SD^26^. In our study, it was also found that the ratio of secondary infection in patients with SD was significantly higher than patients with DF. Our findings confirmed that the positive detection rate of IgA in patients with secondary infection was significantly higher than patients with primary infection. This indirect evidence also supported the association of IgA and disease severity. In our work, the total ratio of secondary infection among the enrolled patients was high (28.2%), and it is a little unexpected in a non-endemic country, which may be related to co-circulation of multiple DENV serotypes in Guangdong province in recent years^27^.

Our study has some limitations. Firstly, the sample distribution was incomparable (at patient level) during days 1–3 and >7 days (Table S2). The percentage of DF patients during days 1–3 and >7 days were both less than 50%, and the percentage of SD patients during days 1–3 was only 12%. So, only the data during 4–7 days after the onset of the illness were used to analyze the association between occurrence of DENV-IgA and the severity of disease. Secondly, the IgA positive rate in urine for all patients is 59%, and nearly half of the patients were IgA negative throughout the course of the illness. Moreover, the IgA detection results for the same patients varied at different times, and this inconsistency may be accountable for sampling time, fluid therapy and some other uncertain factors. So, urinary IgA alone may not be a good diagnostic marker for severe dengue infections. In addition, the majority of the patients were infected with DENV serotype 1, and only a limited number of samples were collected from patients infected with other serotypes.

In conclusion, our results demonstrate that IgA is detected more frequently in the urine samples from patients with SD than patients with DF and its presence may be a warning sign for clinical management.

## Methods

### Ethical approval

The study was approved and supervised by the ethics committee of Guangzhou Eighth People’s Hospital (Reference Number 20100835). All experiments were performed in accordance with approved relevant guidelines and regulations. The informed consent was obtained from all subjects.

### Study design

A total of 262 serial urine samples were obtained from 78 hospitalized patients in Guangzhou Eighth People’s Hospital in China. All patients were laboratory confirmed and grouped into DF or SD according to the criteria defined by the 2009 WHO/TDR guidelines^2^. Meanwhile, serum samples were collected from all patients during the acute phrase of illness (within 7 days after the onset of fever) for DENV genome detection by using the DENV serotyping real-time PCR kit (DaAn, China). DENV infection was confirmed by the presence of NS1 antigen in acute sera using the enzyme-linked immunosorbent assay (Diagnostic Kit for Dengue Virus NS1 Antigen, WANTAI, China). Dengue-specific IgM and IgG antibodies in acute sera were determined by capture ELISA (Panbio, Australia), and the infection type (primary or secondary infection) was defined as previous described^28^. In addition, 20 urine samples collected in 2013 from healthy donors were also included as negative control.

### Detection of DENV-specific IgA antibodies

The IgA antibody detection in urine samples was carried out using ASSURE^®^ Dengue IgA Rapid test (MP Diagnostics) according to the recommended protocol. Urine samples were centrifuged at 400 g for 5 min and the supernatant was used for IgA detection. The intensity of IgA signal was recorded as 0.2, 0.5, 1, 2, 3, respectively using an intensity scale provided with the kit.

### Statistical analysis

The OpenEpi version 2.3.1 software was used for statistical analysis. Continuous variables were compared by Student’s t-test, and categorical variables were assessed using the Fisher exact test. Differences were considered significant at p < 0.05.

## Additional Information

**How to cite this article**: Zhao, H. *et al.* Dengue Specific Immunoglobulin A Antibody is Present in Urine and Associated with Disease Severity. *Sci. Rep.* **6**, 27298; doi: 10.1038/srep27298 (2016).

## Supplementary Material

Supplementary Information

## Figures and Tables

**Table 1:** Characteristics of patients infected by DENV*.

	DF (n = 53)No. (%)	SD (n = 25)No. (%)	TotalNo. (%)	P value^a^
Gender				
Male	36 (67.9)	14 (56)	50 (64.1)	0.321
Female	17 (32.1)	11 (44)	28 (35.9)	
Age, years, mean ± SD	38.8 ± 16.7	61.4 ± 20.7	46.0 ± 20.9	<0.001
Infection type				
primary	45 (84.9)	11 (44)	56 (71.8)	<0.001
secondary	8 (15.1)	14 (56)	22 (28.2)	
AKI^b^, No. (%)	11 (20.8)	16 (64.0)	27 (34.6)	<0.001
Serotype				
DENV-1	41 (77.4)	21 (84.0)	62 (79.5)	
DENV-2	3 (5.7)	1 (4.0)	4 (5.1)	
DENV-3	3 (5.7)	0 (0)	3 (3.8)	
DENV-4	4 (7.5)	0 (0)	4 (5.1)	

^*^Data are from 78 patients recruited from Guangzhou Eighth People’s Hospital.

^a^P values are for the comparison of dengue fever (DF) and severe dengue (SD).

^b^AKI denotes acute kidney injury.

**Table 2:** Summary of the detection of IgA among DF and SD patients at different time points of the disease.

No. of days of fever	DF	SD
	Positive/Total no. (%)	Positive/Total no. (%)
1–3	3/16 (18.8)	1/3 (33.3)
4–7	15/46 (32.6)	13/19 (68.4)
>7	16/24 (66.7)	18/18 (100)
Total	24/53 (45.3)	22/25 (88)

**Table 3:** Comparison between IgA detection in the urine of patients with primary infection and secondary infection*.

IgA detection	Infection type	
	Primary	Secondary
Positive	13	13
Negative	43	9

^*^The chi square analysis showed that there was a significant increase of IgA detection rate in the urine from patients with secondary infection compared with primary infection (*P* = 0.0038).
