# Supplementary material for: Dengue Specific Immunoglobulin A Antibody is Present in Urine and Associated with Disease Severity
Source: Sci Rep. 2016 Jun 2;6:27298. doi: 10.1038/srep27298 (PMC4890044; doi:10.1038/srep27298)
Supplement: Supplementary Information [file srep27298-s1.pdf]

Dengue Specific Immunoglobulin A Antibody was Present in Urine and Associated with Disease Severity

Hui Zhao, Shuang Qiu, Wen-Xin Hong, Ke-Yu Song, Jian Wang, Hui-Qin Yang, Yong-Qiang Deng, Shun-Ya Zhu, Fu-Chun Zhang, Cheng-Feng Qin

TABLE S1 Results of IgA detection in urine samples.

**Patient no.**	**Age (year)**	**Gender**	**Classification**	**Days after onset of illness**	**Serotype**	**Infection type**	**AKI**	**IgA (intensity scale)**
1	23	M	DF	5	DENV-1	Primary	+	-
				7				-
				8				-
				10				-
2	41	M	DF	4	-	Primary	-	-

3	55	M	DF	4	DENV-1	Primary	+	-
				7				-
4	38	M	DF	6	DENV-1	Primary	-	-
5	43	M	DF	1	DENV-1	Primary	-	-
				4				0.2
				6				-
				8				-
6	74	M	DF	5	DENV-1	Primary	+	-
				7				-
				9				-
7	30	M	DF	3	DENV-1	Primary	-	-
8	34	M	DF	7	DENV-1	Primary	-	3
9	21	M	DF	6	DENV-2	Primary	-	-

				8				-
10	22	M	DF	5	DENV-1	Primary	-	-
11	27	M	DF	5	DENV-1	Primary	-	-
12	42	F	DF	5	DENV-1	Primary	-	-
				6				-
13	18	M	DF	4	DENV-1	Primary	-	-
14	42	F	DF	5	DENV-1	Primary	-	1
15	36	M	DF	4	DENV-1	Primary	-	-
16	27	M	DF	5	DENV-1	Primary	-	-
17	25	F	DF	5	DENV-1	Primary	-	-
18	26	M	DF	5	DENV-1	Primary	-	-
19	16	M	DF	5	DENV-1	Primary	-	2
20	58	M	DF	5	DENV-1	Primary	+	-

				6				-
				8				1
				9				2
21	15	F	DF	5	DENV-1	Primary	-	-
				7				-
				8				-
22	30	M	DF	4	DENV-1	Primary	-	-
				5				-
				6				-
				7				-
23	23	M	DF	5	DENV-1	Secondary	-	-
				6				0.5
				7				0.5

				8				0.2
				9				1
				10				-
				11				0.5
				12				-
				13				-
				14				-
24	40	M	DF	4	DENV-1	Primary	-	-
				6				-
				7				-
				8				2
25	34	F	DF	5	-	Primary	+	-
26	47	F	DF	5	DENV-1	Primary	-	-

				6				0.2
				7				1
				8				2
27	42	F	DF	3	DENV-1	Primary	+	2
				4				2
				5				-
				6				-
				7				1
				8				2
28	23	M	DF	5	DENV-1	Primary	-	-
				6				-
				7				-
				8				-

				9				-
				10				-
				11				0.2
				12				0.5
29	63	M	DF	5	DENV-2	Primary	+	-
				6				-
				7				-
				8				0.5
				9				1
30	38	M	DF	4	DENV-1	Primary	-	-
				5				-
				6				-
				7				0.5

				8				1
				9				0.5
				10				-
				11				-
				12				-
31	24	F	DF	4	DENV-1	Primary	-	-
				5				-
				7				-
32	61	F	DF	5	DENV-1	Primary	+	2
				6				-
				7				-
				8				-
				9				-

				11				0.2
				12				0.2
33	16	M	DF	5	DENV-1	Primary	-	-
				6				-
				7				-
				8				-
34	60	F	DF	10	DENV-4	Primary	+	0.2
35	56	M	DF	3	DENV-3	Secondary	+	-
				5				-
36	32	M	DF	6	DENV-4	Primary	-	-
				7				0.5
37	71	F	DF	7	DENV-4	Secondary	+	-
38	46	M	DF	4	DENV-4	Secondary	-	0.2

				5				0.2
				6				-
				7				-
				8				0.5
39	39	F	DF	9	DENV-1	Secondary	-	0.5
				12				-
40	24	F	DF	10	DENV-3	Secondary	-	-
				11				-
				12				-
				13				-
				14				-
41	36	M	DF	7	DENV-3	Primary	-	-
				8				-

42	45	M	SD	6	DENV-1	Primary	-	-
43	69	F	SD	9	DENV-1	Secondary	+	3
44	45	M	SD	8	-	Secondary	-	0.2
45	45	F	SD	6	-	Primary	-	-
46	39	M	SD	7	DENV-1	Secondary	-	-
				10				1
47	35	M	SD	5	DENV-1	Primary	-	-
				6				-
				8				0.2
48	28	M	SD	6	DENV-1	Secondary	+	0.2
49	72	F	SD	3	DENV-1	Primary	+	-
50	33	F	SD	4	DENV-1	Primary	-	2
51	26	M	SD	5	DENV-1	Secondary	-	-

				6				0.2
				7				0.5
				8				0.5
52	40	M	SD	4	DENV-1	Primary	+	0.2
				5				-
				6				-
				7				0.5
				8				-
				9				-
				10				0.2
				11				0.2
				12				0.5
53	60	F	SD	3	DENV-1	Primary	+	0.5

				5				-
				6				-
				7				1
				9				1
				10				0.5
				11				-
				12				-
				13				0.2
				14				1
54	61	F	SD	6	DENV-1	Secondary	-	3
55	70	F	SD	5	DENV-1	Primary	-	1
56	74	F	SD	8	-	Secondary	+	0.2
				9				0.2

				10				0.5
				11				0.5
57	59	F	DF	3	DENV-1	Secondary	-	-
				4				-
				5				1.0
				6				2.0
				7				3.0
				8				3.0
58	19	M	DF	3	DENV-1	Primary	-	-
				4				-
				5				-
59	49	M	DF	1	DENV-2	Primary	-	-
				2				-

				3				-
60	30	F	DF	3	DENV-1	Primary	-	0.2
				4				-
				5				-
				6				-
61	78	F	DF	2	DENV-1	Secondary	-	-
				3				-
				4				-
				5				-
				6				0.2
				7				1.0
				8				1.0
62	46	M	DF	3	DENV-1	Primary	-	-

				4				-
				5				-
				6				-
				7				-
				8				0.5
63	30	M	DF	3	DENV-1	Primary	-	-
				4				-
64	27	M	DF	3	DENV-1	Primary	-	-
65	88	F	SD	2	DENV-2	Primary	+	-
				8				3.0
66	34	M	DF	2	DENV-1	Primary	-	-
				3				-
				4				-

				7				-
67	26	F	DF	1	DENV-1	Primary	-	-
				2				-
68	73	M	DF	3	DENV-1	Primary	-	-
				4				-
				5				-
				7				0.2
				8				1.0
69	66	M	DF	2	DENV-1	Primary	-	0.2
				3				-
				4				-
				5				0.2
70	85	F	SD	5	DENV-1	Secondary	+	0.2

				6				0.2
				7				0.5
				8				0.5
				9				0.5
71	76	M	SD	5	DENV-1	Secondary	+	0.2
				6				1
				8				3
				9				3
				10				3
				12				3
				14				3
72	78	M	SD	7	DENV-1	Secondary	+	3
				9				3

				10				3
				11				3
				12				3
				13				3
73	58	M	SD	6	DENV-1	Secondary	+	0.2
				7				0.5
				8				1
				9				1
74	99	F	SD	8	DENV-1	Secondary	+	1
				9				1
				11				3
				12				3
				14				3

75	75	M	SD	4	DENV-1	Secondary	+	-
				5				-
				6				-
				7				-
				8				0.2
				9				0.5
				10				0.2
				11				0.5
76	75	M	SD	7	DENV-1	Primary	+	0.5
				8				0.5
				10				0.5
				11				1
				12				0.5

				13				0.2
77	85	M	SD	7	DENV-1	Secondary	+	3
				8				3
78	74	M	SD	6	DENV-1	Primary	+	-
				7				-
				8				-
				9				-
				10				-
				12				0.2
				14				0.5

Table S2 | The distribution of patients with dengue fever (DF) or severe dengue (SD) at different time points of the disease.

No. of days of fever	DF (n=53) No. (%)	SD (n=25) No. (%)
1-3	16 (30.2)	3 (12)
4-7	46 (86.8)	19 (76)
>7	24 (45.3)	18 (72)
